# The Good, the Bad and the Tick

**DOI:** 10.3389/fcell.2019.00079

**Published:** 2019-05-15

**Authors:** Alejandro Cabezas-Cruz, Agustin Estrada-Peña, Jose de la Fuente

**Affiliations:** ^1^UMR BIPAR, INRA, ANSES, Ecole Nationale Vétérinaire d'Alfort, Université Paris-Est, Maisons-Alfort, France; ^2^Faculty of Veterinary Medicine, University of Zaragoza, Zaragoza, Spain; ^3^SaBio, Instituto de Investigación en Recursos Cinegéticos IREC-CSIC-UCLM-JCCM, Ciudad Real, Spain; ^4^Department of Veterinary Pathobiology, Center for Veterinary Health Sciences, Oklahoma State University, Stillwater, OK, United States

**Keywords:** ticks, *Theileria* spp., *Anaplasma phagocytophilum*, cancer, networks, malignant transformation

How tick-borne pathogens (TBPs) could help us understand cancer? The diversity of pathogens transmitted by ticks is higher than that of any other known arthropod vector and includes protozoa (e.g., *Babesia* spp. and *Theileria* spp.), bacteria (e.g., intracellular *Rickettsia* spp. and extracellular *Borrelia* spp.), viruses (e.g., Tick-borne encephalitis (TBE) and Crimean-Congo hemorrhagic fever (CCHF) virus), helminths (e.g., *Cercopithifilaria*) and, although less known, fungi (e.g., *Dermatophilus*) (Otranto et al., 2013; Brites-Neto et al., 2015; de la Fuente et al., 2017). TBPs have complex life cycles that involve vertebrate hosts and the ticks. Intracellular TBP infection triggers cellular and molecular responses that change host cell physiology in fundamental ways. Within vertebrate host cells, the apicomplexan parasites *Theileria parva* and *Theileria annulata* activate molecular pathways that result in increased production of reactive oxygen species (ROS), cell immortalization, cancer and host death. In contrast, infection by the rickettsia *Anaplasma phagocytophilum* inhibits apoptosis, block the production of ROS and results in a self-limiting infection that rarely is lethal for the host. *Theileria* spp. and *A. phagocytophilum* modulates host cell response by inducing transcriptional reprogramming of their vertebrate host cells, leukocytes. Transcriptional reprogramming is induced by pathogen-encoded effector proteins that modify host epigenetic pathways that affect not only gene transcription but also protein levels. The complexity of molecular pathways modulated by TBP infection in vertebrate host cells parallel that of cancer which offers a unique opportunity for comparative studies to understand complex health problems such as cancer. Identification of differences between the molecular pathways hijacked by *Theileria* spp. and *A. phagocytophilum* with those leading to non-infectious cancer will provide insights into proteins, pathways and biological processes (BP) associated with malignant transformation.

This hypothesis is based in the following rationality: (i) *Theileria* spp. (Cheeseman and Weitzman, 2015), *A. phagocytophilum* (Sinclair et al., 2014) and oncogenic factors (González-Herrero et al., 2018) behave as “epigenators” (Berger et al., 2009; Cheeseman and Weitzman, 2015) because they have the potential to trigger intracellular signaling pathways that lead to changes in chromatin status and gene expression, (ii) transcriptional reprograming and proteome modulation are hallmarks of infection by *Theileria* spp. (Kinnaird et al., 2013) and *A. phagocytophilum* (de la Fuente et al., 2005; Lee et al., 2008), and oncogenesis (González-Herrero et al., 2018), (iii) transcriptional reprograming and proteome modulation in *Theileria* spp. and *A. phagocytophilum* infections and oncogenesis are associated with similar molecular and cellular processes including apoptosis (Borjesson et al., 2005; Brown and Attardi, 2005; Hayashida et al., 2010; Ayllón et al., 2015), metabolic reprograming (Medjkane and Weitzman, 2013; Yu et al., 2018; Cabezas-Cruz et al., 2019; Masui et al., 2019), oxidative stress and ROS production (IJdo and Mueller, 2004; Medjkane et al., 2014; Takaki et al., 2019) among others. To compare the cell response to *Theileria* spp. and *A. phagocytophilum* infections and carcinogens we propose the combination of quantitative proteomics and network analysis (Figure 1). Networks of proteins and BPs clustered in *Emerging Biological Pathways* (i.e. network modules resulting from the clustering of proteins and BPs in response to different stimuli) can represent the topology of the specific cell response to *Theileria* spp. and *A. phagocytophilum* infection and exposure to carcinogens. The significance of proteins and processes can be then ranked and hierarchized by indexes representing the centrality of proteins and processes in the networks.

**Figure 1:**
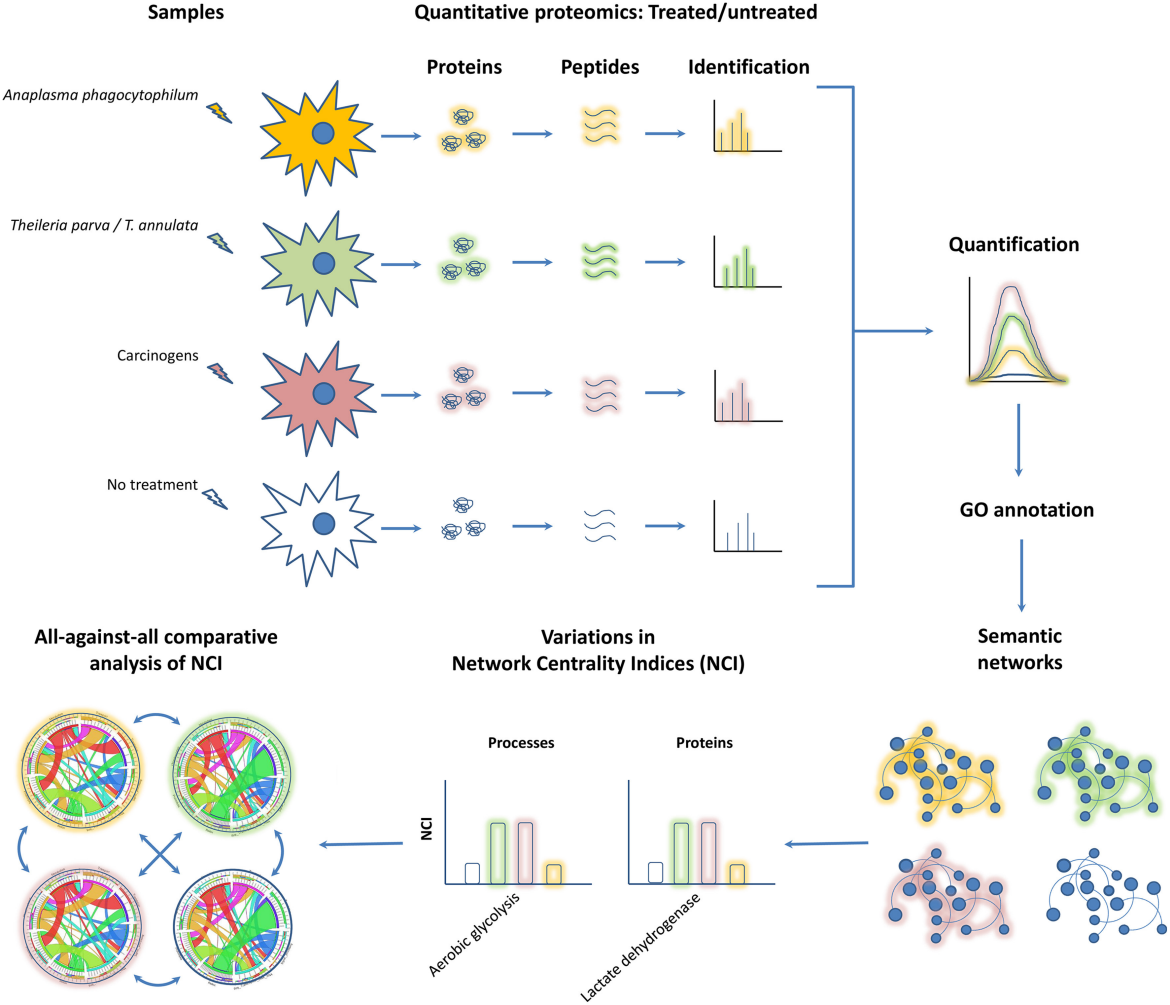
Integration of quantitative proteomics with network analysis to elucidate proteins and BPs key to malignant transformation. The figure displays a workflow previously proposed for the characterization of cell response to *A. phagocytophilum* infection (Estrada-Peña et al., 2018). Samples can be prepared using different stimuli, in this case, a non-transforming bacterium (i.e., *A. phagocytophilum*, colored in orange), a transforming apicomplexan parasite (i.e., *T. parva* and/or *T. annulata*, colored in green) and carcinogens (i.e., know chemical compounds capable of causing cancer, colored in brown). Following standard quantitative proteomics and GO annotation, the names of proteins (nodes type 1) are linked to the biological process (BP) (nodes type 2) in which that protein participates using bipartite networks. The links between nodes type 1 and 2 have strength equivalent to protein expression levels (expressed as normalized Peptide-Spectrum Matches) in treated and untreated cells (no colored). In these networks, BPs with many proteins connected to it and proteins with high relative expression will play a key role in keeping the structure of the network. After comparing with untreated cells, three types of phenotypic changes would be uncovered, those related with infectious transformation (in networks resulting from *Theileria* spp. infection), non-infectious transformation (in networks resulting from cells exposed to carcinogens), and non-transforming infection (in networks resulting from *A. phagocytophilum* infection). Finally, comparing the centrality indices of proteins and BPs in cells treated with carcinogens and those infected with *Theileria* spp and *A. phagocytophilum* will reveal key players in non-infectious transformation. Likewise, comparing the centrality indices of proteins and BPs in cells infected with *Theileria* spp. and *A. phagocytophilum* will provide proteins and BPs critical to infectious transformation. Other comparisons following this logic can provide further insights on infection and cancer.

## Tick-Borne Pathogens As Models In Cancer Research

Infection-induced malignant transformation accounted for 17.8% (1.9 million cases) of the global cancer burden in the year 2002 (Parkin, 2006). The contribution of infectious diseases to cancer epidemiology increased in 2008 to ~2 million new cancer cases attributable to infection with viruses, platyhelminthes, and bacteria (Oh and Weiderpass, 2014). The loss of cellular identity and the transformation of normal into tumor cells is a central and challenging problem in cellular biology. Major advances have been made in understanding the genetic basis and phenotypic changes underlining the continuum from normal cell to tumor cell to malignant transformation (Hanahan and Weinberg, 2000, 2011; Vogelstein et al., 2013). DNA mutations are observed in all types of cancer (Vogelstein et al., 2013). A significant proportion of cancer patients, however, do not have known coding driver mutations and several non-coding mutations affecting not gene function but gene transcription have been identified in cancer (Fredriksson et al., 2014; Zhang et al., 2018; Reyna et al., 2019). In consequence, the attention has been shifted to phenotypic changes induced by aberrant gene expression that also drive tumor and malignant transformation (Guo et al., 2017; Karki et al., 2017; Parfett and Desaulniers, 2017).

In contrast to virus-induced tumorigenesis that include DNA mutations in somatic cells (Ji et al., 2014), *Theileria*-induced tumorigenesis in bovines does not involve changes in DNA sequence (Cheeseman and Weitzman, 2015; Tretina et al., 2015). Instead, epigenetic mechanisms underlie phenotypic changes associated with *Theileria*-induced malignant transformation (Cheeseman and Weitzman, 2015). *Theileria* is consider as a good model to study the molecular basis of phenotypic changes associated with transformation (Cheeseman and Weitzman, 2015; Marsolier et al., 2015). Comparing the *T. annulata* genome with that of *Toxoplasma gondii* (as a control of intracellular and non-transforming apicomplexan parasite), Marsolier et al. (2015) identified 33 *Theileria*-specific proteins among which they found a homolog of mammalian Pin1, a Peptidyl Prolyl Isomerase that regulates cell proliferation, pluripotency, and survival (Marsolier et al., 2015). The human homolog of Pin1 is overexpressed in breast cancer, increases the transcriptional activity of c-Jun and promotes tumor growth (Wulf et al., 2001). It turned out that by interacting and inducing the degradation of FBW7 protein, which degrades c-Jun, *Theileria*'s Pin1 induces c-Jun accumulation and activates the oncogenic c-Jun pathway which in turn promote transformation (Marsolier et al., 2015; Fernandes et al., 2018).

Thus, comparing the genomes of transforming (i.e., *Theileria*) and non-transforming (i.e., *Toxoplasma*) parasites proved a valid strategy for the identification of Pin1 as a protein relevant in cell transformation and tumor growth (Wulf et al., 2001; Marsolier et al., 2015). Oncogenic viruses such as Kaposi's Sarcoma Herpesvirus also activates c-Jun activity in host cells via virus-encoded proteins (An et al., 2004; Hamza et al., 2004; Xie et al., 2005). However, the complexity of the mechanisms leading tumor transformation is revealed by the fact that infections by non-transforming pathogens (e.g., Reovirus and *Staphylococcus aureus*) also induce and activate c-Jun transcriptional activity (Clarke et al., 2001; Borjesson et al., 2005). *Staphylococcus aureus* infection induces *JUN* (the gene encoding for c-Jun) expression in neutrophils (Borjesson et al., 2005), and staphylococcal α-Toxin activates c-Jun by inducing phosphorylation of its serine 73 (Moyano et al., 2018). Reoviruses can also activate c-Jun activity (Clarke et al., 2001) and were even proposed as cancer therapy (Harrington et al., 2010). Another more general example is that in both *Theileria*-induced and non-infectious neoplastic transformation, apoptosis is inhibited (Fernald and Kurokawa, 2013; Dasgupta et al., 2016). Apoptosis inhibition is therefore considered a hallmark of cancer (Hanahan and Weinberg, 2000, 2011). However, *A. phagocytophilum* infection also inhibits apoptosis but, as mentioned above, it does not result in malignant cell immortalization. One conclusion can be reached from these simple comparisons; c-Jun activation, or apoptosis inhibition, alone do not suffice to transform normal cells in tumor cells. What other pathway or pathways have to be modified in a cell to become a tumor cell? A comprehensive comparison between the timing and totality of cell molecular pathways modulated by *Theileria* spp., *A. phagocytophilum* and carcinogens may provide an integrative view of the molecular pathways leading to malignant transformation.

## Semantic Networks To Find The Keywords

In graph theory, a network is a set of nodes that are connected by edges (also known as links). In networks representing food webs (Dunne et al., 2013) or host-parasite interactions (Lafferty et al., 2006; Estrada-Peña et al., 2015), nodes are the organisms and the links represent interactions between them. The directionality and strength of the interactions can be measured as the “weight of interaction” (e.g., the number of times a parasite has been found on a host). We proposed to build “semantic networks” (Estrada-Peña et al., 2018) to capture the changes in cell response induced by different stimuli, *Theileria* spp., *A. phagocytophilum* and carcinogens (Figure 1). In such framework, two type of nodes can be distinguished [i.e., proteins with Gene Ontology (GO) annotation (Villar et al., 2014) and BPs], and the links between them would be the participation of proteins in one or more BPs (Estrada-Peña et al., 2018). In this regard, semantic networks are directed because a ‘source’ (i.e., the protein) is linked to a “destination” (i.e., the BP). In addition, the links have weight equivalent to the protein levels measured by quantitative proteomics and the Degree of each node is proportional to either the protein level or the sum of links reaching a BP.

Initially, semantic networks were used to describe the global cell transformation in response to *A. phagocytophilum* infection (Estrada-Peña et al., 2018). The results demonstrated that the resulting interactions between proteins and BP can be used to calculate the centrality indices of each node of the network (Estrada-Peña et al., 2018). Centrality indices are fundamental measures of the structure of a network and account for intimate changes in the relative importance of key functions. In addition, centrality indices can be used to identify both proteins and BP that are “central” and therefore occupy prominent positions in the cellular response to different stimuli. The argument here is that centrality indices (e.g., Degree centrality, Weighted Degree, and Betweenness Centrality) are powerful indicators of subtle changes in the proteome, which could be missed when standard protein representation analysis is used. Comparison of centrality indices between *A. phagocytophilum*-infected and non-infected human and tick cells revealed (i) that infection by this pathogen rewires the network of cell processes and changes the relative importance of biological pathways and (ii) that tick and human cells respond differently to *A. phagocytophilum* infection (Estrada-Peña et al., 2018). More importantly, the ras-related protein Rab14, with a high centrality in infected tick cells, was selected for functional validation by gene knockdown. Rab14 knockdown resulted in a significant decrease in *A. phagocytophilum* infection levels, suggesting that *A. phagocytophilum* increases the relative importance of Rab14 in the proteome to facilitate infection (Estrada-Peña et al., 2018). The identification of Rab14 as a key protein in *A. phagocytophilum* infection shows that in addition to reveal the global cell response to stimuli, semantic networks can be also used to identify individual proteins that change the relative importance of different BPs and can be validated in further laboratory experiments (Estrada-Peña et al., 2018).

Network analysis has been used previously to study cancer progression and reversal (Parikh et al., 2014), to prioritize rare mutations in protein-coding and non-coding genomic regions (Fredriksson et al., 2014; Zhang et al., 2018; Reyna et al., 2019), to study how *PIK3CA* mutations interact with others components of luminal-breast cancer cell signaling network and predict clinical outcomes (McGee et al., 2017) among others. Most network approaches to study cancer use protein-protein interactions where the nodes are proteins and the links between them represent physical protein-protein interactions (Ozturk et al., 2018). In signaling networks, nodes are also proteins but the links represent signaling relations between them (McGee et al., 2017). Other approaches explicitly violate or relax rules of gene and/or protein interactions and allows for biological noise and uncertainty that are expected to occur in tumor cells (Creixell et al., 2015). Our approach is different to those previously reported in two fundamental ways: (i) semantic networks (Estrada-Peña et al., 2018) connect nodes using GO terms which are broader in scope than pathways (Creixell et al., 2015) or protein-protein interactions, and (ii) the links between nodes are weighted based in experimentally-determined protein levels. Thus, semantic networks have the potential to identify not only key BPs, but also those proteins with the higher contribution to that BP in response to the selected stimuli. These two properties, protein-BP connectivity and weighted contribution of proteins result in Emerging Biological Pathways unique to the stimuli in question (e.g., *Theileria* spp., *A. phagocytophilum* and carcinogens). The characterization of key proteins and BPs may lead to the identification of fundamental processes involved in carcinogenesis, with possible implication in disease prevention and control.

## Author Contributions

AC-C, AE-P, and JdF: conceived the idea, drafted the manuscript, reviewed and accepted the manuscript in its current form.

### Conflict of Interest Statement

The authors declare that the research was conducted in the absence of any commercial or financial relationships that could be construed as a potential conflict of interest.

## References

[B1] An J. Sun Y. Rettig M. B. (2004). Transcriptional coactivation of c-Jun by the KSHV-encoded LANA. Blood 103, 222–228. 10.1182/blood-2003-05-1538 12969971

[B2] Ayllón N. Villar V. Galindo R. C. Kocan K. M. Šíma R. López J. A.. (2015). Systems biology of tissue-specific response to *Anaplasma phagocytophilum* reveals differentiated apoptosis in the tick vector *Ixodes scapularis*. PLoS Genet. 11:e1005120. 10.1371/journal.pgen.1005120 25815810 PMC4376793

[B3] Berger S. L. Kouzarides T. Shiekhattar R. Shilatifard A. (2009). An operational definition of epigenetics. Genes. Dev. 23, 781–783. 10.1101/gad.1787609 19339683 PMC3959995

[B4] Borjesson D. L. Kobayashi S. D. Whitney A. R. Voyich J. M. Argue C. M. Deleo F. R. (2005). Insights into pathogen immune evasion mechanisms: *Anaplasma phagocytophilum* fails to induce an apoptosis differentiation program in human neutrophils. J. Immunol. 174, 6364–6372. 10.4049/jimmunol.174.10.6364 15879137

[B5] Brites-Neto J. Duarte K. M. Martins T. F. (2015). Tick-borne infections in human and animal population worldwide. Vet. World. 8, 301–315. 10.14202/vetworld.2015.301-315 27047089 PMC4774835

[B6] Brown J. M. Attardi L. D. (2005). The role of apoptosis in cancer development and treatment response. Nat. Rev. Cancer. 5, 231–237. 10.1038/nrc1560 15738985

[B7] Cabezas-Cruz A. Espinosa P. Alberdi P. de la Fuente J. (2019). Tick-pathogen interactions: the metabolic perspective. Trends Parasitol. 35, 316–328. 10.1016/j.pt.2019.01.006 30711437

[B8] Cheeseman K. Weitzman J. B. (2015). Host-parasite interactions: an intimate epigenetic relationship. Cell Microbiol. 17, 1121–1132. 10.1111/cmi.12471 26096716

[B9] Clarke P. Meintzer S. M. Widmann C. Johnson G. L. Tyler K. L. (2001). Reovirus infection activates JNK and the JNK-dependent transcription factor c-Jun. J. Virol. 75, 11275–11283. 10.1128/JVI.75.23.11275-11283.2001 11689607 PMC114712

[B10] Creixell P. Reimand J. Haider S. Wu G. Shibata T. Vazquez M.. (2015). Pathway and network analysis of cancer genomes. Nat. Methods 12, 615–621. 10.1038/nmeth.3440 26125594 PMC4717906

[B11] Dasgupta A. Nomura M. Shuck R. Yustein J. (2016). Cancer's Achilles' Heel: Apoptosis and Necroptosis to the Rescue. Int. J. Mol. Sci. 18:E23. 10.3390/ijms18010023 28025559 PMC5297658

[B12] de la Fuente J. Antunes S. Bonnet S. Cabezas-Cruz A. Domingos A. G. Estrada-Peña A.. (2017). Tick-pathogen interactions and vector competence: identification of molecular drivers for tick-borne diseases. Front. Cell. Infect. Microbiol. 7:114. 10.3389/fcimb.2017.00114 28439499 PMC5383669

[B13] de la Fuente J. Ayoubi P. Blouin E. F. Almazán C. Naranjo V. Kocan K. M. (2005). Gene expression profiling of human promyelocytic cells in response to infection with *Anaplasma phagocytophilum*. Cell. Microbiol. 7, 549–559. 10.1111/j.1462-5822.2004.00485.x 15760455

[B14] Dunne J. A. Lafferty K. D. Dobson A. P. Hechinger R. F. Kuris A. M. Martinez N. D.. (2013). Parasites affect food web structure primarily through increased diversity and complexity. PLoS Biol. 11:e1001579. 10.1371/journal.pbio.1001579 23776404 PMC3679000

[B15] Estrada-Peña A. de la Fuente J. Ostfeld R. S. Cabezas-Cruz A. (2015). Interactions between tick and transmitted pathogens evolved to minimise competition through nested and coherent networks. Sci. Rep. 5:10361. 10.1038/srep10361 25993662 PMC4438610

[B16] Estrada-Peña A. Villar M. Artigas-Jerónimo S. López V. Alberdi P. Cabezas-Cruz A.. (2018). Use of graph theory to characterize human and arthropod vector cell protein response to infection with *Anaplasma phagocytophilum*. Front. Cell. Infect. Microbiol. 8:265. 10.3389/fcimb.2018.00265 30123779 PMC6086010

[B17] Fernald K. Kurokawa M. (2013). Evading apoptosis in cancer. Trends Cell Biol. 23, 620–633. 10.1016/j.tcb.2013.07.006 23958396 PMC4091735

[B18] Fernandes R. Ferreira S. Botelho M. C. (2018). Commentary: theileria parasites secrete a prolyl isomerase to maintain host leukocyte transformation. Front. Med. 5:120. 10.3389/fmed.2018.00120 29755983 PMC5934426

[B19] Fredriksson N. J. Ny L. Nilsson J. A. Larsson E. (2014). Systematic analysis of non-coding somatic mutations and gene expression alterations across 14 tumor types. Nat. Genet. 46, 1258–1263. 10.1038/ng.3141 25383969

[B20] González-Herrero I. Rodríguez-Hernández G. Luengas-Martínez A. Isidro-Hernández M. Jiménez R. García-Cenador M. B.. (2018). The making of leukemia. Int. J. Mol. Sci. 19:E1494. 10.3390/ijms19051494 29772764 PMC5983781

[B21] Guo Y. Nie Q. MacLean A. L. Li Y. Lei J. Li S. (2017). Multiscale modeling of inflammation-induced tumorigenesis reveals competing oncogenic and oncoprotective roles for inflammation. Cancer Res. 77, 6429–6441. 10.1158/0008-5472.CAN-17-1662 28951462

[B22] Hamza M. S. Reyes R. A. Izumiya Y. Wisdom R. Kung H. J. Luciw P. A. (2004). ORF36 protein kinase of Kaposi's sarcoma herpesvirus activates the c-Jun N-terminal kinase signaling pathway. J. Biol. Chem. 279, 38325–38330. 10.1074/jbc.M400964200 15247271

[B23] Hanahan D. Weinberg R. A. (2000). The hallmarks of cancer. Cell 100, 57–70. 10.1016/S0092-8674(00)81683-9 10647931

[B24] Hanahan D. Weinberg R. A. (2011). Hallmarks of cancer: the next generation. Cell 144, 646–674 10.1016/j.cell.2011.02.013 21376230

[B25] Harrington K. J. Vile R. G. Melcher A. Chester J. Pandha H. S. (2010). Clinical trials with oncolytic reovirus: moving beyond phase I into combinations with standard therapeutics. Cytokine Growth Factor Rev. 21, 91–98. 10.1016/j.cytogfr.2010.02.006 20223697 PMC3915505

[B26] Hayashida K. Hattori M. Nakao R. Tanaka Y. Kim J. Y. Inoue N.. (2010). A schizont-derived protein, TpSCOP, is involved in the activation of NF-kappaB in Theileria parva-infected lymphocytes. Mol. Biochem. Parasitol. 174, 8–17. 10.1016/j.molbiopara.2010.06.005 20540970

[B27] IJdo J. W. Mueller A. C. (2004). Neutrophil NADPH oxidase is reduced at the *Anaplasma phagocytophilum* phagosome. Infect Immun. 72, 5392–5401. 10.1128/IAI.72.9.5392-5401.2004 15322037 PMC517486

[B28] Ji X. Zhang Q. Du Y. Liu W. Li Z. Hou X.. (2014). Somatic mutations, viral integration and epigenetic modification in the evolution of hepatitis B virus-induced hepatocellular carcinoma. Curr. Genomic 15, 469–480. 10.2174/1389202915666141114213833 25646075 PMC4311391

[B29] Karki R. Man S. M. Kanneganti T. D. (2017). Inflammasomes and Cancer. Cancer Immunol. Res. 5, 94–99. 10.1158/2326-6066.CIR-16-0269 28093447 PMC5593081

[B30] Kinnaird J. H. Weir W. Durrani Z. Pillai S. S. Baird M. Shiels B. R. (2013). A bovine lymphosarcoma cell line infected with theileria annulata exhibits an irreversible reconfiguration of host cell gene expression. PLoS ONE. 8:e66833. 10.1371/journal.pone.0066833 23840536 PMC3694138

[B31] Lafferty K. D. Dobson A. P. Kuris A. M. (2006). Parasites dominate food web links. Proc. Natl. Acad. Sci. U.S.A. 103, 11211–11216. 10.1073/pnas.0604755103 16844774 PMC1544067

[B32] Lee H. C. Kioi M. Han J. Puri R. K. Goodman J. L. (2008). *Anaplasma phagocytophilum*-induced gene expression in both human neutrophils and HL-60 cells. Genomics 92, 144–151. 10.1016/j.ygeno.2008.05.005 18603403

[B33] Marsolier J. Perichon M. DeBarry J. D. Villoutreix B. O. Chluba J. Lopez T.. (2015). *Theileria* parasites secrete a prolyl isomerase to maintain host leukocyte transformation. Nature 520, 378–382. 10.1038/nature14044 25624101 PMC4401560

[B34] Masui K. Onizuka H. Cavenee W. K. Mischel P. S. Shibata N. (2019). Metabolic reprogramming in the pathogenesis of glioma: update. Neuropathology 39, 3–13. 10.1111/neup.12535 30609184

[B35] McGee S. R. Tibiche C. Trifiro M. Wang E. (2017). Network analysis reveals a signaling regulatory loop in the PIK3CA-mutated breast cancer predicting survival outcome. Genom. Proteom. Bioinforma. 15, 121–129. 10.1016/j.gpb.2017.02.002 28392480 PMC5414713

[B36] Medjkane S. Perichon M. Marsolier J. Dairou J. Weitzman J. B. (2014). *Theileria* induces oxidative stress and HIF1α activation that are essential for host leukocyte transformation. Oncogene 33, 1809–1817. 10.1038/onc.2013.134 23665677

[B37] Medjkane S. Weitzman J. B. (2013). A reversible Warburg effect is induced by Theileria parasites to transform host leukocytes. Cell Cycle. 12, 2167–2168. 10.4161/cc.25540 23803730 PMC3755061

[B38] Moyano A. J. Racca A. C. Soria G. Saka H. A. Andreoli V. Smania A. M.. (2018). c-Jun Proto-oncoprotein plays a protective role in lung epithelial cells exposed to staphylococcal α- and Toxin. Front. Cell. Infect. Microbiol. 8:170. 10.3389/fcimb.2018.00170 29888211 PMC5981160

[B39] Oh J. K. Weiderpass E. (2014). Infection and cancer: global distribution and burden of diseases. Ann. Glob. Health 80, 384–392. 10.1016/j.aogh.2014.09.013 25512154

[B40] Otranto D. Dantas-Torres F. Brianti E. Traversa D. Petrić D. Genchi C.. (2013). Vector-borne helminths of dogs and humans in Europe. Parasit Vectors 6:16. 10.1186/1756-3305-6-16 23324440 PMC3564894

[B41] Ozturk K. Dow M. Carlin D. E. Bejar R. Carter H. (2018). The emerging potential for network analysis to inform precision cancer medicine. J. Mol. Biol. 430, 2875–2899. 10.1016/j.jmb.2018.06.016 29908887 PMC6097914

[B42] Parfett C. L. Desaulniers D. (2017). A Tox21 approach to altered epigenetic landscapes: assessing epigenetic toxicity pathways leading to altered gene expression and oncogenic transformation *in vitro*. Int. J. Mol. Sci. 18:E1179. 10.3390/ijms18061179 28587163 PMC5486002

[B43] Parikh A. P. Curtis R. E. Kuhn I. Becker-Weimann S. Bissell M. Xing E. P.. (2014). Network analysis of breast cancer progression and reversal using a tree-evolving network algorithm. PLoS Comput. Biol. 10:e1003713. 10.1371/journal.pcbi.1003713 25057922 PMC4109850

[B44] Parkin D. M. (2006). The global health burden of infection-associated cancers in the year 2002. Int. J. Cancer 118, 3030–3044. 10.1002/ijc.21731 16404738

[B45] Reyna M. A. Haan D. Paczkowska M. Verbeke L. P. C. Vazquez M. Kahraman A. (2019). PCAWG drivers and functional annotation group, ICGC/TCGA pan-cancer analysis of whole genomes. pathway and network analysis of more than 2,500 whole cancer genomes. bioRxiv 2019:385294 10.1101/385294

[B46] Sinclair S. H. Rennoll-Bankert K. E. Dumler J. S. (2014). Effector bottleneck: microbial reprogramming of parasitized host cell transcription by epigenetic remodeling of chromatin structure. Front. Genet. 5:274. 10.3389/fgene.2014.00274 25177343 PMC4132484

[B47] Takaki A. Kawano S. Uchida D. Takahara M. Hiraoka S. Okada H. (2019). Paradoxical roles of oxidative stress response in the digestive system before and after carcinogenesis. Cancers 11:E213. 10.3390/cancers11020213 30781816 PMC6406746

[B48] Tretina K. Gotia H. T. Mann D. J. Silva J. C. (2015). *Theileria*-transformed bovine leukocytes have cancer hallmarks. Trends Parasitol. 31, 306–314. 10.1016/j.pt.2015.04.001 25951781

[B49] Villar M. Popara M. Ayllón N. Fernández de Mera I. G. Mateos-Hernández L. Galindo R. C.. (2014). A systems biology approach to the characterization of stress response in *Dermacentor reticulatus* tick unfed larvae. PLoS ONE 9:e89564. 10.1371/journal.pone.0089564 24586875 PMC3931811

[B50] Vogelstein B. Papadopoulos N. Velculescu V. E. Zhou S. Diaz L. A. Kinzler K. W. (2013). Cancer genome landscapes. Science 339, 1546–1558 10.1126/science.1235122 23539594 PMC3749880

[B51] Wulf G. M. Ryo A. Wulf G. G. Lee S. W. Niu T. Petkova V.. (2001). Pin1 is overexpressed in breast cancer and cooperates with Ras signaling in increasing the transcriptional activity of c-Jun towards cyclin D1. EMBO J. 20, 3459–3472. 10.1093/emboj/20.13.3459 11432833 PMC125530

[B52] Xie J. Pan H. Yoo S. Gao S. J. (2005). Kaposi's sarcoma-associated herpesvirus induction of AP-1 and interleukin 6 during primary infection mediated by multiple mitogen-activated protein kinase pathways. J. Virol. 79, 15027–15037. 10.1128/JVI.79.24.15027-15037.2005 16306573 PMC1316010

[B53] Yu X. Ma R. Wu Y. Zhai Y. Li S. (2018). Reciprocal regulation of metabolic reprogramming and epigenetic modifications in cancer. Front. Genet. 9:394. 10.3389/fgene.2018.00394 30283496 PMC6156463

[B54] Zhang W. Bojorquez-Gomez A. Velez D. O. Xu G. Sanchez K. S. Shen J. P. (2018). A global transcriptional network connecting non-coding mutations to changes in tumor gene expression. Nat. Genet. 50, 613–620. 10.1038/s41588-018-0091-2 29610481 PMC5893414

